# The association of rs25487 of the *XRCC1* gene and rs13181 of the *ERCC2* gene polymorphisms with the ovarian cancer risk

**DOI:** 10.17305/bb.2024.11314

**Published:** 2024-12-13

**Authors:** Tatiana Zavarykina, Maria Kapralova, Polina Lomskova, Aleksandra Asaturova, Grigory Khabas, Lyailya Kayumova, Dmitry Khodyrev, Irina Pronina, Maya Sannikova, Svetlana Khokhlova

**Affiliations:** 1 N.M. Emanuel Institute of Biochemical Physics of Russian Academy of Sciences, Moscow, Russia; 2 “B.I. Kulakov National Medical Research Center of Obstetrics, Gynecology, and Perinatology”, Ministry of Health of the Russian Federation, Moscow, Russia; 3 Sechenov First Moscow State Medical University (Sechenov University), Moscow, Russia; 4 Federal Scientific and Clinical Center of Specialized Types of Medical Care and Medical Technologies, Federal Medical and Biological Agency of the Russian Federation, Moscow, Russia; 5 Institute of General Pathology and Pathophysiology, Moscow, Russia; 6 Yaroslav-the-Wise Novgorod State University, Novgorod, Russia

**Keywords:** Ovarian cancer, OC, DNA repair, *XRCC1 gene*, *ERCC2 gene*, polymorphism, odds ratio, OR, rs25487, rs13181

## Abstract

Ovarian cancer (OC) is the most lethal gynecological cancer worldwide. DNA damage plays an important role in cancer development, and the proteins encoded by *XRCC1* and *ERCC2* are important components of the DNA repair system. This study aimed to examine the relationship between the rs25487 *XRCC1* and rs13181 *ERCC2* polymorphisms and the risk of OC development in women from the Moscow region. DNA was isolated from the blood of 129 healthy donors and tissues and blood samples from 125 patients with OC and studied using real-time PCR. An increase in odds ratios (OR) was obtained for OC tissue and blood for both *T* (OR ═ 1.46, 95% confidence interval [CI] ═ 1.22–1.76, *P* ═ 0.00005), and for *T/T* of rs25487 *XRCC1*. The most significant OR values were found for the *T/T* genotype using the codominant model (OR ═ 2.11, 95% CI ═ 1.44–3.07, *P* ═ 0.00006) and dominant model (OR ═ 3.13, 95% CI ═ 1.44–6.79, *P* ═ 0.0025) for the pooled blood and tissue groups. For rs13181 *ERCC2,* differences were observed for the *T/G* genotype in OC tissues (OR ═ 0.69, 95% CI ═ 0.51–0.92, *P* ═ 0.011) in the codominant model. In this study, the association of allele *T* and genotypes of rs25487 *XRCC1* and *T/G* of rs13181 *ERCC2* with OC was shown. Our results indicate that these polymorphisms may be involved in the pathogenesis of OC and are promising for further studies on therapeutic applications in OC.

## Introduction

Approximately 324,000 new cases of ovarian cancer (OC) are diagnosed annually, accounting for 6.6% of all cancer cases in women. It ranks seventh in morbidity and sixth in mortality worldwide among women [1]. OC is characterized by a long asymptomatic course and rapid spread of the tumor process; advanced disease is diagnosed in 70%–80% of cases [2, 3]. The clinical outcomes of OC depend on the stage at diagnosis as well as the optimal volume of surgical treatment and drug therapy. The development of malignant tumors is influenced by lifestyle, environmental, and genetic factors. The latter includes the functioning of tumor suppressor genes and the activation of oncogenes in the cell, which are often associated with the functioning of the DNA repair system. *BRCA1* and *BRCA2* tumor suppressor genes are the most important for OC. Germline mutations in these genes are responsible for hereditary variants of OC, whereas OC somatic mutations are associated with the development of OC in some cases. However, *BRCA1* and *BRCA2* mutations occur in 10%–15% of OC cases [4]. Another important genetic factor is the DNA repair system. Accumulation of DNA damage leads to mutations, chromosomal restructuring, and malignant transformation. Therefore, DNA repair is one of the most important systems for preventing carcinogenesis. In addition, acquired and hereditary defects in the DNA repair system can predispose patients to malignant neoplasms [5]. The proliferation of cells with defects in the repair system may increase the frequency of mutations and enhance the genetic instability [6, 7]. Most DNA damage is repaired by excision systems, such as base excision repair (BER) and nucleotide excision repair (NER).

BER is required for the point removal of DNA bases damaged by oxidative and alkylating agents. The XRCC1 protein is an integral BER protein and is encoded by the *XRCC1* gene [8]. Polymorphisms in genes can affect the function of proteins and are relatively common in the population compared to mutations. One of the most studied and essential polymorphisms in the *XRCC1* gene is rs25487 (*Gln399Arg*), which manifests as a T to C substitution in the gene (A to G at 1196 position of mRNA). This leads to an amino acid change in the encoded protein at position 399: replacement of glutamine (*Gln*) with arginine (*Arg*). This substitution occurs in the PARP binding domain of the XRCC1 protein, which may affect complex assembly and repair efficiency [9, 10]. The frequency of the reference allele in the world is *T* ═ 0.344, and that of the alternative allele is *C* ═ 0.656. In different populations these frequencies vary: *T* ═ 0.115–0.372; *C* ═ 0.628–0.885.

Polymorphism rs25487 of *XRCC1* is associated with the risk of developing various types of cancer, including breast [11], lung [12], pancreatic [13], and nasopharyngeal cancers [14]. In a meta-analysis of 26 publications, including 6979 patients with colorectal cancer and 11,470 healthy people, a statistically significant association between this marker and the risk of the disease was obtained (allele *A* (*T*) vs allele *G* (*C*): odds ratio (OR) ═ 1.13, *P* ═ 0.008; *Gln/Gln* (*T/T*) vs *Arg/Arg (C/C)*: OR ═ 1.24, *P* ═ 0.015) [15]. In a study by Meza-Espinoza et al. [16] in the Northeastern Mexican Population, an association with colorectal cancer was found for the allele *A* (*T*) (allele *G* (*C*) vs allele *A* (*T*); OR ═ 1.48, *P* ═ 0.034) and for the genotype *A/A* (*T/T*) in a codominant model (*A/A* (*T/T*) vs *G/G (C/C)*; OR ═ 3.11, *P* ═ 0.031). In addition, the polymorphism *Arg399Gln* of the *XRCC1* gene with the risk of developing colorectal cancer in the study by Kabzinski et al. [17] revealed that *Arg/Gln* (*T/C*) genotype increased the risk of colorectal cancer (OR ═ 2.481, 95% confidence interval [CI]: 1.745–3.529, *P* < 0.0001). The study by Malisic and Krivokuca [18], conducted on the Serbian population, found that the *Arg (C)* allele of rs25487 in the *XRCC1* gene is associated with an increased risk of OC (OR ═ 2.64, *P* < 0.01) compared to the *Gln (T)* allele. However, the results obtained for the different populations were inconsistent. In a study by Verma et al. [19] in the Indian population, there was no statistically significant association between this marker and the risk of OC development (OR ═ 1.5, *P* ═ 0.271). In a study of the Brazilian population, no differences in the genotype distribution frequency of rs25487 in the *XRCC1* gene were found [20]. The only recent case-control study showed borderline significance for this marker (OR ═ 1.43, *P* ═ 0.063) in a dominant inheritance model in the Chinese population [21].

Another variant of DNA excision repair is the NER. One of the key genes in this system is *ERCC2* or *XPD*. It encodes an ATP-dependent DNA helicase, which is an important component of the TFIIH protein complex in NER repair and is responsible for the initial recognition of DNA damage [22]. One of the most studied polymorphisms in the *ERCC2* (*XPD*) gene is rs13181. It is located in 23th exon of the *ERCC2* gene and is manifested as the replacement of T to G, resulting in a change in the amino acid *Lys* to *Gln* in the C-terminal part of the protein. These polymorphisms can cause changes in protein function, thereby affecting DNA repair activity. The *G* (*Gln)* allele is associated with low repair ability and increased chromatid breaks frequency [23]. Worldwide, the frequency of the reference allele is *T* ═ 0.643, the alternative allele is *G* ═ 0.357, varying in different populations: *T* ═ 0.62–0.92, *G* ═ 0.37–0.08.

Some polymorphisms of the *ERCC2* gene are associated with a high risk of developing various types of cancer, such as breast cancer [24, 25], lung cancer [26], hepatocellular cancer [27], and leukemia [28]. Furthermore, *ERCC2* gene expression can be increased in tumor tissues [29]. Several studies have shown that the *G* (*Gln)* allele of rs13181 is associated with an increased risk of developing lung cancer [26], breast cancer [30], and bladder cancer [31]. In a study by Michalska et al. [32], the association of the rs13181 polymorphism with the risk of OC development was observed in the Polish population. However, there is some inconsistency in the relationship between the rs13181 of *ERCC2* gene and the risk of OC development. In a meta-analysis by Li et al. [33], a significant association between *ERCC2* gene rs13181 polymorphism and increased risk of OC was revealed, although Zhang and Zhang [34] concluded that this polymorphism may not be associated with the risk of OC. In some cases, such discrepancies may be due to the differences in the populations studied.

Therefore, the study of genes and polymorphisms predisposing to the development of OC in different populations is an important issue in the study of the pathogenesis of this disease for better understanding of it. However, there are few studies on the association between the single nucleotide polymorphisms rs25487 in the *XRCC1* gene and rs13181 in the *ERCC2* gene and OC in Caucasians. Therefore, it is difficult to draw a representative conclusion about their contribution to the development of this disease, even using a meta-analysis approach. The aim of this study was to investigate the relationship between rs25487 in the *XRCC1* gene and rs13181 in the *ERCC2* gene and the risk of OC development in women from the Moscow region of Russia

## Materials and methods

### Study population

The study was conducted in compliance with the principles of voluntariness and confidentiality in accordance with the Federal Law “on the Fundamentals of Protecting the Health of Citizens in the Russian Federation” and the 1964 Declaration of Helsinki and its subsequent amendments. Informed consent was obtained from all the participants included in the study. The biological samples of OC patients were obtained from the Kulakov National Medical Research Center of Obstetrics, Gynecology, and Perinatology of the Ministry of Health of Russia. Blood samples from healthy donors were obtained from Sechenov First Moscow State Medical University.

**Figure 1.**
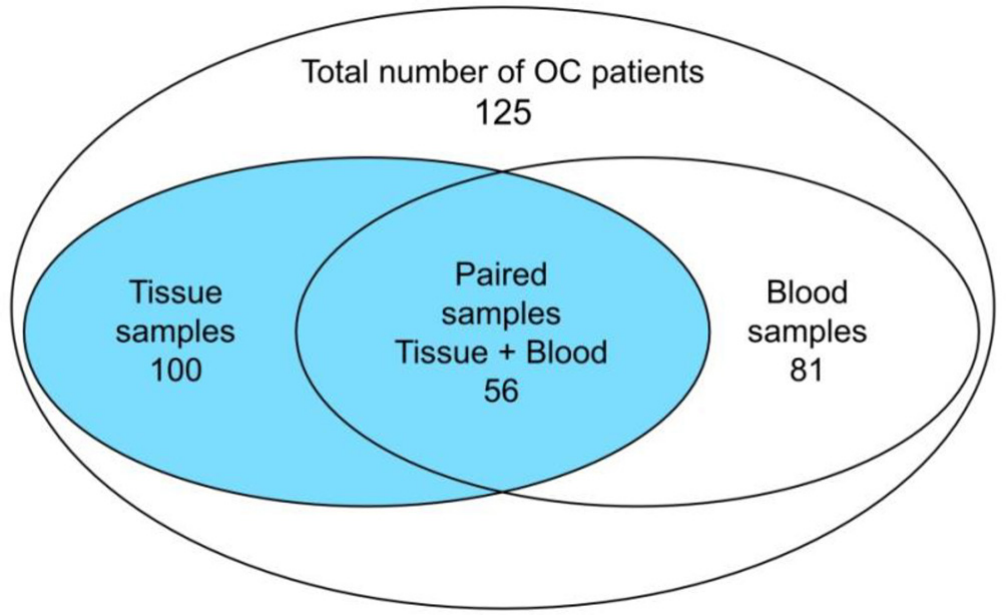
**Scheme of OC patients’ samples.** Colored zone—tissue samples in pooled group of tissue and blood. OC: Ovarian cancer.

The criteria for inclusion of patients in the study were a morphologically confirmed diagnosis of OC, satisfactory general condition, and normal function of hematopoiesis, kidneys, and liver. The diagnosis and histological form of OC were established by histological examination at the Kulakov National Medical Research Center of Obstetrics, Gynecology, and Perinatology. In this study, samples from 264 people were studied: 129 healthy Caucasian female donors with a median age of 48 years (min 23–max 70), and 125 Caucasian OC patients: tumor tissue from 100 patients with OC (stage Ia–IV) with a median age of 52.4 years (32–75 years) and blood samples of the patients (81 people with a median age of 49.6 (28–75]), including 56 samples from the patients whose tissues were collected (paired samples) (Figure 1). Most patients (86%) had a histological form of serous OC. Blood and tissue samples were obtained before the start of chemotherapy, and tumor tissue samples were collected during the primary debulking surgery. All samples were stored at 80 ^∘^C.

### Analysis of rs25487 *XRCC1* and rs13181 *ERCC2* polymorphisms

DNA was isolated from the blood and tissue samples using a Diatom DNA Prep 400 reagent kit (Isogen Laboratory, Russia). The polymorphisms rs25487 in the *XRCC1* gene and rs13181 in the *ERCC2* gene were determined using real-time PCR with fluorescent allele-specific probes on a CFX96 Touch Real-Time System thermal cycler (Bio-Rad, USA). Allelic discrimination was performed using Bio-Rad CFX Manager software. The sequences and annealing temperatures (Ta) of primers and probes used in this study are listed in Table 1.

**Table 1:** Conditions of the analysis of *rs25487* of the *XRCC1* gene and *rs13181* of the *ERCC2* gene

**Polymorphism**	Primers and probes^*^	**T_a_^#^, ^∘^C/length, bp**
*XRCC1 rs25487*	F: GCTCCTCTCAGTAGTCTG R: CTGGCATCTTCACTTCTG FAM: CCTTACCTCTGGGAGGGC VIC: CCTTACCTCCGGGAGGGC	65.7/283
*ERCC2 rs13181*	F: CTGACTTCATAAGACCTTCTAG R: TCTCCCTTTCCTCTGTTC FAM: TCTATCCTCTTCAGCGTCTCC VIC: TCTATCCTCTGCAGCGTCTCC	63.7/217

^*^F and R are primers, where F is forward and R is reverse; FAM and VIC are DNA probes labeled with the corresponding fluorescent dyes: The minor variant of the polymorphism corresponds to the VIC probe. ^#^T_a_ refers to the annealing temperature.

PCR was carried out in 10 µL of the reaction mixture with the following composition: 70 mM Tris-HCl, pH 8.8, 16.6 mM ammonium sulfate, 0.01% Tween-20, 2 mM magnesium chloride, 200 nM of each dNTP, 500 nM primers (Evrogen, Russia), 250 nM fluorescent probes (DNK-Sintez, Russia), 1.5 units Taq DNA polymerase (Evrogen). Conditions for amplification of DNA fragments: 95 ^∘^C for 20 s; 40 cycles: 95 ^∘^C for 10 s, Ta for 30 s, and 72 ^∘^C for 30 s.

### Ethical statement

The study was approved by the Biomedical Research Ethics Committee of Kulakov National Medical Research Center of Obstetrics, Gynecology, and Perinatology of the Ministry of Health of Russia, protocol No. 03, 26/02/2021.

### Statistical analysis

Statistical analysis was carried out using Statistica 8.0 (StatSoft) and IBM SPSS Statistics 27.0.1.0. Analysis of the relationship between polymorphisms and the risk of development of OC was carried out in groups of tissue samples and blood, as well as in the united group of tissue and blood, if appropriate. Fischer’s exact test was used to compare the frequencies of alleles between groups of tissue samples and blood of OC patients. To compare allele and genotype Pearson’s χ^2^ test was used. The obtained results were checked for compliance with the Hardy–Weinberg equilibrium for each polymorphism. The relationship between the studied alleles, genotypes, and disease risk was determined using regression analysis in IBM SPSS Statistics 27.0.1.0, determining the OR and 95% CI. Differences were considered statistically significant at *P* < 0.05.

## Results

The genotype frequencies of rs25487 in the *XRCC1* gene and rs13181 in the *ERCC2* gene were obtained for healthy donors and patients with OC. In the group of OC patients, for each marker, the OR calculation was performed in groups of blood and tissue samples. The results were compared between these two groups, and if they were not statistically different, the OR was calculated in the combined “blood + tissue” group to increase the statistical significance of the study.

### Polymorphism rs25487 of *XRCC1* gene

The frequency distribution of genotypes for rs25487 in the *XRCC1* gene is presented in Table 2. The distribution of genotype frequencies of the polymorphism in the control group corresponded to Hardy–Weinberg equilibrium (*P* ═ 0.34). No differences were found between the blood and tissue subgroups of patients with OC when comparing the distribution of allele frequencies (Fisher’s exact test, *P* ═ 0.91 for all blood and tissue samples; *P* ═ 0.53, paired blood and tissue samples from each patient).

**Table 2:** Frequency distribution of genotypes of *rs25487* of the *XRCC1* gene in OC patients and healthy donors

**Gene**	**Genotype**	**Genotype frequencies**		
		**Control (*n* ═ 129)**	**Tumor tissue (*n* ═ 100)**	**Blood (*n* ═ 81)**
*XRCC1 rs25487*	*T/T*	0.078	0.200	0.210
	*T/C*	0.395	0.490	0.444
	*C/C*	0.527	0.310	0.346

The OR for OC development was calculated. The results for each allele of rs25487 of *XRCC1* gene when comparing data for blood samples from healthy donors with tissue and blood samples from patients with OC are presented in Table 3. An increase in the risk of OC development for carriers of the *T* allele of rs25487 of *XRCC1* gene was found (OR ═ 1.45, *P* ═ 0.00016 for tissue samples; OR ═ 1.28, *P* ═ 0.0218 for blood samples). The differences in the allele frequencies obtained for tissue and blood samples from OC patients were insignificant, as were the genotype frequencies. Therefore, to increase the statistical significance of the study, the analysis was performed in a pooled group of blood and tissue samples from different patients (125 people) (Figure 1). As a result, an increase in the OR for OC development in carriers of the *T* allele was also observed (OR ═ 1.46, *P* ═ 0.00005) for the pooled tissue and blood group (*n* ═ 125). For carriers of the *T/T* genotype, an increase in the OR was also found in carriers of the T/T genotype. The most significant values were obtained when the codominant model was used for tissue (OR ═ 2.10, *P* ═ 0.00019) and for the pooled tissue and blood group, *n* ═ 125 (OR ═ 2.11, *P* ═ 0.00006) (Table 4). Higher OR were observed for tissue (OR ═ 2.48, *P* ═ 0.0009) and pooled tissue and blood group (OR ═ 2.46, *P* ═ 0.00049) using the recessive model. Using the dominant model (*T/T* vs *C/C*+*T/C*), the pooled tissue and blood group yielded an OR of 3.13, *P* ═ 0.0025. Close OR values were obtained for blood samples in the dominant model (*T/T*+*T/C* vs *C/C*, OR ═ 3.16, *P* ═ 0.0060).

**Table 3:** The odds ratio for the risk of OC development for alleles of *rs25487* of the *XRCC1* gene

**Allele**	OR^*^	95% CI^**^	**χ^2^**	***P***
*Tumor tissue*				
Allele *T*	1.45	1.20–1.77	14.26	0.00016
Allele *C*	0.69	0.57–0.84	14.26	0.00018
*Blood*				
Allele *T*	1.28	1.04–1.58	5.26	0.0218
Allele *C*	0.71	0.57–0.87	5.26	0.00098
*Pooled group of tissue and blood (n ═ 125)*				
Allele *T*	1.46	1.22–1.76	16.55	0.00005
Allele *C*	0.68	0.57–0.82	16.55	0.00005

*OR: Odds ratio; ^#^CI: Confidence interval; OC: Ovarian cancer.

**Table 4:** The odds ratio of the risk of OC development for the genotypes of *rs25487* of the *XRCC1* gene using codominant, dominant, and recessive models

**Inheritance model**	**Genotype *rs25487 XRCC1***	OR^*^	95% CI^**^	**χ^2^**	***P***
Codominant	*Tumor tissue*				
	*T/T*	2.10	1.41–3.13	13.93	0.00019
	*C/T*	1.54	1.17–2.04	9.43	0.00213
	*C/C*	0.48	0.32–0.71	13.93	0.00019
	*Blood*				
	*T/T*	1.94	1.28–2.94	10.26	0.00136
	*T/C*	1.40	1.05–1.88	5.19	0.0227
	*C/C*	0.52	0.34–0.78	10.26	0.00136
	*Pooled group of tissue and blood (*n* ═ 125)*				
	*T/T*	2.11	1.44–3.07	15.98	0.00006
	*T/C*	1.53	1.18–2.00	10.15	0.0014
	*C/C*	0.48	0.33–0.69	15.98	0.00006
Dominant	*Tumor tissue*				
	*T/T*	Reference		7.41	0.0065
	*T/C+C/C*	2.98	1.32–6.69		
	*Blood*				
	*T/T*	Reference		7.56	0.0060
	*T/C+C/C*	3.16	1.37–7.31		
	*Pooled group of tissue and blood (n ═ 125)*				
	*T/T*	Reference		9.14	0.0025
	*T/C+C/C*	3.13	1.44–6.79		
Recessive	*Tumor tissue*				
	*T/T*	Reference		10.98	0.0009
	*T/C+C/C*	2.48	1.44–4.29		
	*Blood*				
	*T/T*	Reference		6.68	0.0010
	*T/C+C/C*	2.11	1.19–3.75		
	*Pooled group of tissue and blood (*n* ═ 125)*				
	*T/T*	Reference		12.17	0.00049
	*T/C+C/C*	2.46	1.47–4.10		

*OR: Odds ratio; ^#^CI: Confidence interval; OC: Ovarian cancer.

Tumor grade data were available for a number of patients with serous OC (*n* ═ 55). The majority had high-grade OC (*n* ═ 45), while a small proportion had a rarer low-grade OC variant (*n* ═ 10). We found that these subgroups of patients retained OR values similar to those in the overall group of patients, which was less pronounced for low-grade OC owing to the small number of patients. For the subgroup with high-grade OC, the data were repeated for the overall group of patients: an increase in the OR value for the *T* allele (OR ═ 1.52, CI 1.19–1.95, *P* ═ 0.0010) and for the *T/T* genotype for all inheritance models (OR ═ 2.28–3.40, *P* ═ 0.013–0.001). However, for patients with low-grade OC, only a tendency toward an increase in OR was observed for the *T* allele (OR ═ 1.47, CI 0.93–2.34, *P* ═ 0.109) and *T/T* genotype in the codominant model (OR ═ 2.028, CI 0.83–4.94, *P* ═ 0.125), and a significant increase in OR was observed for the dominant inheritance model (OR ═ 7.93, CI 1.92–32.83, *P* ═ 0.008).

### Polymorphism rs13181 of *ERCC2* gene

The frequency distribution of genotypes of rs13181 of *ERCC2* gene is presented in Table 5. The frequency of genotypes in the control group corresponded to Hardy–Weinberg equilibrium (*P* ═ 0.40). No differences were found between the blood and tissue groups of patients with OC when comparing the distribution of allele frequencies (Fisher’s exact test, *P* ═ 0.91 for all blood and tissue samples; *P* ═ 0.88, paired blood and tissue samples from each patient).

**Table 5:** Frequency distribution of genotypes of *rs13181* of the *ERCC2* gene in OC patients and healthy donors

**Gene**	**Genotype**	**Genotype frequencies**		
		**Control** **(*n* ═ 129)**	**Tumor tissue** **(*n* ═ 100)**	**Blood** **(*n* ═ 81)**
*ERCC2 rs13181*	*T/T*	0.333	0.440	0.395
	*T/G*	0.543	0.340	0.444
	*G/G*	0.124	0.220	0.161

OC: Ovarian cancer.

The results of the OR calculation for the risk of OC development for rs13181 of *ERCC2* gene are presented in Tables 6 and 7. No differences were observed in the alleles of this polymorphism (Table 6). When considering the genotypes, differences in the OR values for tissue and blood samples were revealed (Table 7). Using the codominant model of inheritance, a statistically significant decrease in OR was found in heterozygotes *T/G* (OR ═ 0.69, *P* ═ 0.011) for tumor tissue samples. However, this relationship was not statistically significant for blood samples. The dominant and recessive models showed a borderline increase in OR for the alternative homozygous *G/G* genotype in tissue samples (*G/G*+*T/G* vs *T/T*: OR ═ 1.57, *P* ═ 0.099; *G/G* vs *T/G*+*T/T*: OR ═ 1.99, *P* ═ 0.0538), but this was not statistically significant in blood samples. For tissue and blood samples of OC patients, there were differences in the genotype frequencies of rs13181 *ERCC2*, so it was not reasonable to combine tissue and blood samples.

**Table 6:** The odds ratio for the risk of OC development for alleles of *rs13181* of the *ERCC2* gene

**Allele**	OR^*^	95% CI^**^	**χ^2^**	***P***
*Tumor tissue*				
Allele *T*	1.003	0.83–1.21	0.001	0.97
Allele *G*	0.997	0.83–1.21	0.001	0.97
Blood				
Allele *T*	1.019	0.83–1.25	0.032	0.86
Allele *G*	0.98	0.80–1.20	0.032	0.86
Pooled group of tissue and blood (*n* ═ 125)				
Allele *T*	1.016	0.85–1.21	0.030	0.86
Allele *G*	0.98	0.82–1.18	0.030	0.86

*OR: Odds ratio; ^#^CI: Confidence interval; OC: Ovarian cancer.

**Table 7:** The odds ratio of the risk of OC development for the genotypes of the *rs13181* of the *ERCC2* gene using codominant, dominant and recessive models

**Inheritance model**	**Genotype** ***rs13181 ERCC2***	OR^*^	95% CI^**^	**χ^2^**	***P***
*Codominant*	*Tumor tissue*				
	*T/T*	1.022	0.71–1.48	0.013	0.91
	*T/G*	0.69	0.51–0.92	6.53	0.011
	*G/G*	0.98	0.67–1.42	0.013	0.91
	*Blood*				
	*T/T*	1.06	0.70–1.60	0.071	0.79
	*T/G*	0.83	0.61–1.12	1.51	0.219
	*G/G*	0.95	0.62–1.43	0.071	0.79
*Dominant*	*Tumor tissue*				
	*T/T*	Reference		2.72	0.099
	*T/G+ G/G*	1.57	0.92–2.69		
	*Blood*				
	*T/T*	Reference		0.82	0.37
	*T/G+ G/G*	0.77	0.43–1.36		
*Recessive*	*Tumor tissue*				
	*T/T+T/G*	Reference		3.72	0.0538
	*G/G*	1.99	0.98–4.03		
	*Blood*				
	*T/T+T/G*	Reference		0.548	0.46
	*G/G*	1.35	0.61–2.98		

*OR: Odds ratio; ^#^CI: Confidence interval; OC: Ovarian cancer.

Consideration of the co-carriage of minor alleles of the *XRCC1* and *ERCC2* polymorphisms was not performed because no reliable association with OC was registered for the alleles of *ERCC2* gene marker. The identified associations for the genotypes of these markers in the codominant model were in different directions; therefore, combining them into one regression model was not possible. The combination of favorable genotypes for the two studied markers also resulted in a reduced risk of developing OC (χ^2^ ═ 19.56, *P* ═ 0.00057).

## Discussion

### The relationship between the rs25487 of the *XRCC1* gene and the risk of OC development

According to the Ensembl.org database (as of August 25, 2024), the alternative allele C of rs25487 in the *XRCC1* gene is correlated with low expression of *XRCC1* mRNA in the ovary and other tissues. Meanwhile, *XRCC1* expression is associated with a higher stage of OC, platinum resistance, an increased risk of death, and worse prognosis [35]. There are a lot of earlier works dedicated to effect of this marker for various applications [36]. In our study, an increase in the OR for OC development in carriers of the *T* allele was observed (OR ═ 1.45, *P* ═ 0.00016 for tissue samples; OR ═ 1.28, *P* ═ 0.0218 for blood samples; OR ═ 1.46, *P* ═ 0.00005 for pooled tissue and blood group, *n* ═ 125). For the *T/T* genotype of rs25487 of *XRCC1*, a statistically significant increase in the risk of development of OC was found in all models of inheritance (OR ═ 2.94–3.16, *P* ═ 0.0065–0.00006). The most significant values were observed when the codominant (OR ═ 2.11, *P* ═ 0.00006) and dominant (OR ═ 3.13, *P* ═ 0.0025) inheritance models were used. Previously, a significant relationship was detected between the *Arg/Gln* (*T/C*) and *Gln/Gln* (*T/T*) genotypes and an increased frequency of induced chromatid breaks as well as between the *Gln* (*T*) allele and spontaneous breaks per cell [37].

The data obtained by Malisic and Krivokuca [18] for the Serbian population showed that the *Arg (C)* allele of the rs25487 marker in the *XRCC1* gene is associated with an increased risk of OC development (OR ═ 2.64, *P* < 0.01). In this study, ovarian carcinoma tissue samples were analyzed, and exfoliated cervical cells were used as controls. The discrepancies with our results may be associated with both population differences and the smaller sample size used in [18], where the Hardy–Weinberg equilibrium was not observed for controls [38]. A meta-analysis [38] showed that in Asians, the *A* (*T*) allele of rs25487 is associated with an increased risk of cancer in the female reproductive system. However, the association with rs25487 disappeared in Asians and appeared in non-Asians after the authors excluded studies deviating from the Hardy–Weinberg equilibrium.

A 2012 study on a population of ethnic Russians found no association between the rs25487 marker in the *XRCC1* gene and the development of OC [39]. The discrepancies in the results of our study are probably related to the characteristics of the study population. In our study, the control group included Caucasian women from the Moscow region, whereas, in the study by Khokhrin et al., the control group included only Russian women who had no interethnic marriages up to the second generation. Simultaneously, the modern population of the European part of Russia (particularly in the Moscow region) consists of a mixed combination with other ethnic groups inhabiting this part of Russia. Since there have been no other studies on the association of this marker with the risk of developing OC in Caucasian populations except mentioned above, subsequent meta-analyses could only be based on these studies, such as the most recent work [40] and earlier work [38].

The results obtained for serous OC patients with tumor grade data suggest that the pathogenesis of both OC types may be similar at a certain stage. In both subgroups with known tumor grade data, an association was found between the carriage of the *T* allele and the development of OC, which was less pronounced for low-grade OC owing to the small number of patients. This observation may indicate the prospects for studying this marker when using PARP inhibitors for both types of serous OC as well as during therapy with Topo I and Topo II topoisomerase inhibitors. The data obtained may be important for selecting chemotherapy for patients with OC, since in some cases, standard neoadjuvant (or adjuvant) platinum-based chemotherapy (carboplatin, paclitaxel) is ineffective, especially in cases of low-grade serous OC. Currently, PARP inhibitors are used only for high-grade serous OC during the maintenance treatment of both primary OC and relapse. Topo I inhibitors are used in combination therapy for relapsed OC and are promising for metronomic chemotherapy [41]. The antitumor antibiotic doxorubicin, which blocks the enzyme topoisomerase II alpha and causes double-stranded DNA breaks, is used in the treatment of primary OC and is the drug of choice for non-platinum agents in combination therapy for relapse.

Topo I inhibitors stabilize the Topo I–DNA cleavage complex at the stage when DNA breaks are formed. The repair of Topo I-induced damage occurs with the participation of the BER system. Simultaneously, cells lacking the key BER protein XRCC1 were hypersensitive to Topo I inhibitors. PARP proteins are important for maintaining cell viability and are involved in various cellular processes, including the repair of single-strand DNA breaks by excision repair. PARP proteins form binding sites for a number of proteins during BER, including XRCC1, and recruit XRCC1 to Topo I-dependent DNA breaks, which in turn recruit tyrosyl-DNA phosphodiesterase (TDP 1), which removes Topo I from DNA. When PARP proteins are inhibited, single-strand DNA breaks are not repaired, leading to replication fork arrest during the S phase of the cell cycle. In addition, PARP1 is able to interact with Topo I and repair Topo I-dependent single-strand DNA breaks. Thus, the effects of topoisomerase I inhibitors may be enhanced in the presence of PARP inhibitors [42, 43]. In addition, there is evidence of the involvement of XRCC1 protein, together with PARP1, in the DNA double-strand break repair system by the microhomology-mediated end joining (MMEJ) method, which is active during the S-phase and G2 phases of the cell cycle [44].

### Relationship between the rs13181 of the ERCC2 gene and the risk of OC development

Early studies in the field of molecular epidemiology found a decrease in NER DNA repair activity for the *Gln/Gln (G/G)* rs13181 genotype of the *ERCC2* gene. It was shown using various methods: by changing the DNA repair capacity [45]; using cytogenetic assays to detect the activity of repair of the induced DNA damage by analyzing the expression of chromosome aberrations specific to the inducing agents [23]; by increasing the aromatic DNA adduct level in minor allele carriers [46]; and decreasing the efficiency of repair of oxidative DNA damage [47]. The latter study by Kabzinski et al. [47] revealed both a decrease in the repair of oxidative DNA damage and an increased risk of colorectal cancer development in carriers of *Gln/Gln* (*G/G*) genotype in one sample of patients. According to the *ensemble.org* database (date 25/08/2024), the alternative allele *G* (*Gln*) of rs13181 of the *ERCC2* gene is correlated with low expression of *ERCC2* mRNA in human tissues. In our study, a reduction in the risk of OC development was observed for heterozygous *T/G* (OR ═ 0.69, *P* ═ 0.011). When using the recessive model, a borderline increase in OR was found for the *G/G* genotype (OR ═ 1.99, *P* ═ 0.054). In a study by Michalska et al. [32], similar results were obtained for the Polish population: a decrease in *Lys/Gln* (*T/G*) genotype (OR ═ 0.41, *P* < 0.0001), and an increased risk of OC (OR ═ 5.01, *P* < 0.0001) for *Gln/Gln* (*G/G*) genotype was observed.

In our study, changes in the rs13181 genotype of the *ERCC2* gene were detected only in tumor tissue samples, but not in blood samples. Michalska et al. [32] used tumor tissue samples. A more pronounced relationship in this work was most likely due to the use of normal ovarian tissue from unrelated women who had never been diagnosed with any tumors as a control. Thus, the genotype *T/G* of this polymorphism is associated with a decreased risk of OC development when comparing not only tumor and healthy ovarian tissues, but also when comparing the blood of healthy women with tumor tissues. However, there were no differences in the blood samples of healthy women and patients with cancer. A shift in the distribution of allele frequencies of this locus during carcinogenesis for various reasons can be assumed, such as deletions and amplifications of chromosomal regions, which are characteristic of the process of cancer development [48, 49].

## Conclusion

In conclusion, an increase in the risk of OC development was found for carriers of the *T* allele and the *T/T* genotype of *XRCC1* gene in both tissue and blood samples of OC patients. For rs13181 of the *ERCC2* gene, the differences were observed only for genotypes and OC tissue samples: a statistically significant decrease in OR was found in heterozygotes *T/G* for tumor tissue samples, and it was not statistically significant in blood samples. Our results indicate that the rs25487 polymorphism in the *XRCC1* gene and the rs13181 polymorphism in the *ERCC2* gene may be involved in the pathogenesis of OC. These findings could be promising for further research into therapeutic applications for OC.

## Data Availability

All data are contained within the article.
